# Pediatric Graves’ disease: management in the post-propylthiouracil Era

**DOI:** 10.1186/1687-9856-2014-10

**Published:** 2014-06-16

**Authors:** Scott A Rivkees

**Affiliations:** 1 Department of Pediatrics, University of Florida College of Medicine, 1600 SW Archer Road – Room R1-118, Gainesville, FL, USA

**Keywords:** Thyroid, Hyperthyroidism, Methimazole, Propylthiouracil, Radioactive iodine, Thyroidectomy, Hepatotoxicity

## Abstract

The most prevalent cause of thyrotoxicosis in children is Graves’ disease (GD), and remission occurs only in a modest proportion of patients. Thus most pediatric patients with GD will need treatment with radioactive iodine (RAI; ^131^I) or surgical thyroidectomy. When antithyroid drugs (ATDs) are prescribed, only methimazole (MMI) should be administered, as PTU is associated with an unacceptable risk of severe liver injury. If remission does not occur following ATD therapy, ^131^I or surgery should be contemplated. When ^131^I is administered, dosages should be greater than 150 uCi/gm of thyroid tissue, with higher dosages needed for large glands. Considering that there will be low-level whole body radiation exposure associated with ^131^I, this treatment should be avoided in young children. When surgery is performed near total or total-thyroidectomy is the recommended procedure. Complications for thyroidectomy in children are considerably higher than in adults, thus an experienced thyroid surgeon is needed when children are operated on. Most importantly, the care of children with GD can be complicated and requires physicians with expertise in the area.

## Graves’ disease

Graves’ disease (GD) is the most common cause of hyperthyroidism in children and is a considerably more pernicious condition than hypothyroidism. The prevalence of GD is 1 in 1,000 adults [1] and is 1 in 10,000 in the pediatric population [2]. GD is due to thyroid gland stimulation by thyroid receptor antibodies [TRAbs; or thyroid stimulating immunoglobulins (TSI)] [3]. Toxic nodules, toxic multinodular goiters, acute and subacute thyroiditis, thyroid hormone ingestion can also cause childhood thyrotoxicosis, but much less commonly than GD [4].

Symptoms of hyperthyroidism include excessive physical activity, tremor, tachycardia, flushing, palpitations, weight loss, accelerated linear growth, reduced bone mineralization, and poor school performance [4]. In childhood GD, ophthalmopathy occurs in less than 50% of patients and is usually mild when present [4].

Because GD spontaneously resolves uncommonly, hyperthyroidism treatment is mandatory. Therapeutic approaches for GD include the antithyroid drugs (ATDs) propylthiouracil (PTU) or methimazole (MMI), radioactive iodine (^131^I), or surgery [4-7]. Each of these modalities has uniquely associated benefits and risks that must be considered when children are treated.

## Antithyroid drugs

ATDs were introduced in the 1940s with thiouracil being the first compound used clinically [8]. Because of the high incidence of toxic reactions associated with thiouracil, this medication was replaced for clinical use by PTU in 1947 [8]. MMI became a treatment option for GD in 1950 [8].

ATDs act by inhibiting oxidation and organic binding of thyroid iodide to impair thyroid hormone production [9]. MMI is ten- to twenty-fold more potent than PTU and has a longer half-life [9]. Importantly, these medications do not cure the hyperthyroid state, rather they palliate the condition. Each of these medications is associated with adverse events that must be considered when prescribed. As such, prior to the initiation of ATD therapy, a back-up plan that takes into account the patient’s age and treatment risks, in the event that a toxic reaction occurs, should be considered.

### Propylthiouracil hepatotoxicity

In 2008, a number of serious complications associated with PTU therapy in children were brought to public attention by Rivkees [10-12]. PTU-induced liver injury at that time accounted for 15% of liver transplants in the United States [13]. From 1990 to 2007, 23 PTU-related liver transplants took place, and 30% of the PTU-related transplant recipients were children. Based on prescribing data, the risk of PTU-induced liver failure leading to transplantation was estimated to be 1 in 2,000 children [2].

Despite a common perception, because PTU-induced liver injury occurs rapidly and is often irreversible, serial monitoring of transaminase levels in a child on PTU, is not viewed to be useful in helping to reduce drug hepatotoxicity risk [2]. As such, the only way to reduce the risks of PTU-related hepatotoxicity is to avoid the use of the medication.

In 2009, Rivkees and Madison recommended that PTU not be used in children, and that PTU be stopped in all children taking the medication in favor of alternative treatments [11]. In April, 2010, the US Food and Drug Administration issued a black box regarding the use of PTU stating that PTU should not be used in children [10], except in special settings, solidifying the notion that the drug should not be used.

### Appropriate limited use of propylthiouracil

Although PTU should be avoided clinically, there is a role for its limited use in special circumstances. PTU can be used when neither prompt ^131^I or surgical treatment are options in a patient who has had a toxic reaction to MMI, and ATD medication is necessary. In this situation, PTU should only be used short-term while plans for ^131^I or surgery are developed.

When PTU is used, patients and guardians need to be informed of the risk of liver failure and to be alert for signs and symptoms of liver abnormalities. These features include pruritus, jaundice, anorexia, light colored stools, dark urine, and abdominal pain. If these problems occur, the patient should immediately stop the medication, a practitioner contacted, and laboratory tests obtained (white blood cell count, bilirubin, alkaline phosphatase, ALT/AST).

### Methimazole

MMI is now the drug-of-choice for GD. Carbimazole, which is a pro-drug that is converted to MMI, can be used in place of MMI in countries where it is available. Although MMI is often prescribed in divided doses over the day, once a day dosing is sufficient [14] and is associated with better compliance than multiple daily doses [15]. The typical MMI dose is 0.2 to 0.5 mg/kg per day, and doses can range from 0.1 to 1.0 mg/kg per day [3,16-20].

MMI is available in 5, 10, and 20 mg tablets. When used in children, the following doses that are fractions of tablets can be used: infants, 1.25 mg per day; 1 to 5 years, 2.5 to 5.0 mg/day; 5 to 10 years, 5 to10 mg/day; and 10 to 18 years, 10 to 20 mg/day. When there is severe hyperthyroidism, one can use double the above doses. Because the hyperthyroid state can be associated with low white cell counts and patients will be treated with a medication that can depress neutrophil levels, it is reasonable to obtain a complete blood count at therapy onset. In addition, we routinely obtain transaminase levels and liver function tests at therapy onset, to assess for premorbid liver disease, as we find that 1% of our patients may have autoimmune hepatitis.

The response to ATDs influencing circulating thyroid hormone levels is not instantaneous, and several months are needed for thyroid hormone levels to normalize [7,14]. Thyroid function tests should thus be obtained monthly after therapy onset. After T4 levels become normal, the MMI doses can be cut by half to maintain euthyroidism [21]. Because TSH levels may take months to normalize, they should not be used to guide changes in medication in early phases of treatment.

Rather than titrating the MMI dose lower when circulating thyroid hormone levels fall, some physicians prefer the block-and-replace approach and add levo-thyroxine while not changing the MMI dose, however, there is a greater risk of adverse events using block and replace vs. dose reduction [21,22]. Recognizing that there is a potential dose–response relationship for some MMI-related complications [23,24], it is preferable to use the lowest MMI dose that achieves control, rather than using the block and replace approach.

Although MMI is the drug of choice for GD, MMI therapy is not without risks. Minor side effects may affect up to 17% of children [25]. The most common minor adverse side effects related to MMI are hives, arthralgia, and neutropenia [25]. Children may also develop major side effects, including Stevens-Johnson syndrome and vasculitis [25]. MMI adverse events most commonly occur within 6 months of therapy onset [25]. Yet, 4% of children will develop adverse events 18 months of MMI therapy, highlighting the need for constant vigilance while on treatment.

Agranulocytosis is another potential serious ATD adverse event and occurs in 0.3% of adults taking PTU or MMI [7,26]. With MMI, agranulocytosis is dose-dependent and is rare at low doses [7,26]. If an individual receiving MMI feels ill, becomes febrile or develops pharyngitis, MMI should be stopped immediately, a practitioner contacted, and a complete blood cell count obtained.

Agranulocytosis typically develops first 3 months of therapy [7,26]. Thus, whereas it is tempting to treat with high-doses of ATD therapy at onset, this approach should be avoided. Rather, relatively lower doses of MMI should be employed initially, and symptoms managed with beta-blockers. Furthermore, the time to normalization of thyroid function tests is only modestly different in individuals treated with high vs. low ATD doses [14].

Although ATDs can be used long-term, reports describe the development of anti-neutrophil-cytoplasmic antibodies (ANCAs), which are associated with vasculitis and may limit prolonged medical therapy of GD [27-29]. In adults up to 15% of individuals treated with PTU, develop ANCAs after 2 years of therapy [27,28]. MMI use is also associated with ANCA-positivity conversion, albeit with a lower incidence than with PTU [27,28].

In the pediatric population, ANCA-mediated disease has been observed with either PTU or MMI [30,31] . Because these antibodies can trigger serious vasculitis events, antithyroid medications should be stopped and definitive therapy considered when ANCA antibodies are detected [32]. To test for this potential problem it is reasonable to perform annual assessment of ANCAs on children on prolonged ATD therapy, i.e. more than two years.

### Duration of therapy

Remission of GD is defined as being biochemically euthyroid or hypothyroid for one year or more after the discontinuation of ATDs. The collective literature indicates that remission rates in children are less than 25% following many years of ATD therapy [33-37] (Table 1).

**Table 1:** Studies of rates of remission related to antithyroid drug use

**Author**	**Date**	**Sample**	Outcome*	**Reference**
Hamburger	1985	262	14%	[54]
Glaser	1997	184	24%	[34]
Glaser	2008	58	29%	[35]
Kaguelidou	2008	154	28%	[36]
Leger	2012	154	48%	[37]

*Remission rate.

Although prolonged ATD treatment will control biochemical hyperthyroidism, it is not clear that prolonged ATD use increases the likelihood of lasting spontaneous remission [38]. In a French study of 94 patients, following treatment for 6 or 18 months, remission rates were 42%and 62% respectively, after two years of treatment [39]. In 52 Spanish patients, following treatment for 12 or 24 months, remission rates were 46% and 54%, respectively, 2 years after cessation of therapy [40]; at 5-years, the relapse rate was 85%. Another study of 134 French patients found no benefit of 18 vs. 43 months of treatment [41]. Thus treating beyond 18 months does not increase remission likelihood in adults.

In the pediatric age group, remission rates range from 20 to 30% following ATDs use for two years or more [18,35,36,42,43]. More than 25 years ago, Lippe and coworkers estimated that 25% of children go into remission for every two years of treatment [44]. Of the 63 patients followed on ATDs, 36 (57%) remitted after an average of four years of therapy [44]. Yet, there were little data to show if the patients who came off ATDs remained in remission [44].

Other large cohort studies of ATD use for many years [33,34] show low remission rates. Of more than 200 children with GD in Minnesota, 25% were in remission after one year; 25% after 2 years; 26% after 4 years; and 15% after 10 years. In addition, 30% of the boys and girls who went into remission had disease recurrence [33].

When 184 pediatric children in California were followed for up to 4 years, the overall remission rate was 23% [34]. After one year of ATDs, 10% were in remission; after 2 years, 14% were in remission; after 3 years, 20%) were in remission; and after 4 years, 23% were in remission.

In a study of children in Argentina, 113 patients received ATDs for prolonged periods [45]. After 10 years of treatment, 33% of patients treated with ATDs went into remission [45].

Most recently, a study performed in France reported that prolonged drug therapy was associated with 50% remission rates in children [37]. One-hundred fifty-four children with GD diagnosed between 1997 and 2002 were examined following treatment with carbimazole. The estimated rates of remission were 20%, 37%, 45%, and 49%, after 4, 6, 8, and 10 years of therapy, respectively [37].

Age-related factors also influence remission likelihood. In a study of 32 prepubertal vs. 68 pubertal children with GD, remission occurred in 17% of prepubertal children treated for 6 years vs. 30% of pubertal children [42]. In another report with pre- and post-pubertal cohorts, remission occurred in 28% of children [46], but the time to remission was three-times longer in the pre-pubertal children than pubertal children [46]. Of note, adverse reactions to ATDs occurred with greater frequency in prepubertal children (71%) than in pubertal (28%) and postpubertal (25%) children [46].

In addition to puberty, TRAb levels and gland size influence remission rates. The efficacy of ATDs is inversely related to circulating levels of TRAbs. Remission rates of GD in adults are about 15% in patients high TRAb levels at diagnosis, and 50% when the pretreatment levels are normal [47]. Large glands at presentation are also associated with much lower remission rates than when gland size is normal [48-50].

### Symptomatic management

In patients treated with ATDs for GD, it may take one or two months until biochemical hyperthyroidism resolves [14]. In the interim, treatment with beta blockers, including propranolol, atenolol or metoprolol, can be used to control GD symptoms. When the patient has asthma, metoprolol is preferred over non-selective beta-blockers, with the patient carefully monitored [51]. When thyroid hormone levels normalize, beta blockers can be stopped.

### Metabolic complications of GD

Increasing evidence shows that GD can be associated metabolic complications. GD can be associated with either hyper- or hypoglycemia at presentation [52,53]. Myopathy has been observed both at initial presentation and when hypothyroidism occurs after therapy [54]. Excessive weight gain has been observed after initiation of therapy, leading to the recommendation that dietary counseling take place when treatment is initiated [55].

## Radioactive iodine

Radioactive iodine uptake by the thyroid is not distinguishable from ordinary iodine, thus radioactive iodine is trapped in thyroid cells [56]. After being taken up by thyroid cells, beta-emissions bring about the destruction of the iodine trapping-cells and those in close proximity [56].

Around ten different isotopes of iodine have been used in medicine. ^123^I is the isotope most frequently used for diagnostic studies of thyroid structure and function [56]. ^121^I has a short half-life (13.3 hrs) and emits x-rays and gamma-photons, but no beta particles. By comparison, ^131^I has a half-life of 6–8 days and emits beta particles and gamma rays.

Radioactive iodine use for thyroid ablation was introduced in the 1940’s at the Massachusetts Institute of Technology and Massachusetts General Hospital [6,57]. When the US Atomic Energy Commission was permitted to supply uranium fission products for medical usage, ^131^I, with an eight-day half-life became available for GD treatment. Because of the intrinsic advantages a longer half-life isotope, ^131^I quickly became the favored iodine isotope for treating thyroid cancer and hyperthyroidism.

### Treatment approach

The goal for ^131^I therapy for GD is to induce hypothyroidism. Radioactive iodine should not be given to cause euthyroidism in children, as this results in partially-irradiated residual thyroid tissue that will be associated with a risk of thyroid neoplasm [58,59]. It has been suggested that dosages delivering 30,000 to 40,000 cGy (rad) to the thyroid are necessary to ablate the thyroid gland [60,61]; however, doses delivering 10,000 to 20,000 cGy to the thyroid are more often used and result in partial or complete destruction of the thyroid [4,62,63].

Typically, administered thyroid doses of 150 uCi/gm (5.5 MBq/gm) generate radiation doses of 12,000 cGy to the thyroid [64]. After ^131^I treatment, radiation exposure to the stomach, marrow, liver, and gonads is about 14, 6.8, 4.8, and 2.5 cGy per organ, respectively, with total body exposure at about 4.0 cGy [64]. Because of fetal risks, ^131^I should not be given to women who are pregnant.

The rate of iodine uptake and the amount of thyroid tissue present influences thyroid destruction potential. Dosages of radioiodine administered are thus based on iodine uptake and gland size using the Quimby-Marinelli equation: dosage (radiation; in Gy) = 90 × oral iodine-131 dose (μCi) × oral 24-hr uptake (%) / gland mass (gm) × 100%). This calculation assumes an effective T/_1/2_ of 6.0 days for ^131^I. Thyroid size is estimated by palpation or ultrasound (ultrasound volume = 0.48 × length × depth × width) [65]. If a patient is taking antithyroid medication, treatment should be stopped 3–5 days before radioactive iodine is administered. After ^131^I administration, the circulating levels of thyroid hormones may increase within 4 to 10 days, as thyroid hormone is released from degenerating follicular cells [66]. Thus if antithyroid medication is discontinued too soon, there can be accumulation of excess thyroid hormone within the gland, leading to an increased risk of thyroid storm following treatment [67].

It usually takes 6 to 12 weeks after ^131^I treatment for the patient to become biochemically euthyroid or hypothyroid. Until then, symptoms of hyperthyroidism can be controlled using beta-blockers [66,68,69]. The use of SSKI or Lugol’s solution one week after ^131^I will also quickly attenuate biochemical hyperthyroidism without adversely affecting the outcome of radioiodine therapy [69].

In as many as 5% of patients, receiving properly calculated dosages, hyperthyroidism will persist after ^131^I. It is recommended that these patients receive a second dose of radioiodine [62], which can be given 6 months after initial therapy. Furthermore, sometimes in those patients with residual thyroid tissues, as indicated by being euthyroid or borderline hypothyroid, hyperthyroidism may recur requiring additional therapy.

Cure rates are higher in patients treated with larger than smaller amounts of ^131^I. When treated with relatively low dosages (50–75 uCi/gm), hyperthyroidism persists in 30 to 50% of adults one year after therapy [70-73]. By comparison, after treatment with higher dosages (150–250 uCi/gm), only 5-10% of patients will remain hyperthyroid at one year [64,74,75].

Radioiodine therapy’s success is influenced by the thyroid gland size and by circulating levels of TRAb. Patients with very large glands (>80 gm) and high TRAb levels have lower responses to ^131^I therapy than patients with smaller glands [63,76-79]. Because of poor response rates with very large glands, thyroidectomy should be considered for individuals with glands greater than 80 gm. But, if ^131^I is used in this setting, patients should be counseled that the risk of needing an additional dose, will be higher than for patients with a smaller gland. Furthermore, surgical removal of very large glands will be associated with greater risk than removal of a smaller gland.

Considering the above, patients treated for GD need to be constantly monitored for progressive thyromegaly, which can change a patients from being an excellent candidate for good ^131^I outcome, to one in which outcomes is poor or associated with increased risks of therapy. Gland size can be grossly estimated by palpation, with the examiner standing behind the patient and feeling each lobe of the thyroid gland with their index fingers to estimate the size of each lobe relative to a teaspoon (5 gm) or tablespoon (15 gm), or multiples thereof. For example if each lobe of the thyroid is about one teaspoon, the estimated gland size is 10 gm. Yet, when there are about two tablespoons or more for each lobe, gland size will be 60 gm or more. When the gland is large, thyroid ultrasound is recommended to more accurately assess gland size. By ultrasound gland size is 0.6 × length × width × depth [80,81].

### Radioactive iodine use in children

Several studies have reported the details of ^131^I therapy for childhood GD [33,82-88]. Children as young as one year old have been treated with ^131^I with excellent results [88,89]. But, treatment of such young children is not common, nor is recommended. ^131^I dosages in children and teenagers have ranged from 100 to 400 uCi/gm of thyroid tissue [4]. Similar to that found in adults, responses to ^131^I therapy are related to gland size and dose. 25 to 40% of children treated with 50–100 uCi of ^131^I per gm of thyroid tissue are hyperthyroid several years after therapy [58]. In children treated with 150–200 uCi of ^131^I per gm thyroid, hyperthyroidism remains in 5-20%, and 60-90% become hypothyroid [4,62,83,89].

Our group analyzed outcomes of the children treated with radioactive iodine therapy to assess the effectiveness of therapy as related to gland size and dose [90]. Following treatment, when treated with 80–120 uCi of ^131^I per gm of thyroid tissue, 28% of children were hyperthyroid, 28% of children were euthyroid, and 42% of children were hypothyroid. Following treatment with 200–250 uCi per gm of thyroid tissue, 37% of children were hyperthyroid and 62% were hypothyroid. Following, treatment with 300–400 uCi per gm of thyroid tissue, 0% of children were hyperthyroid, euthyroid, and 93% were hypothyroid. Comparing these pediatric data with those from adults [63,65,90], thyroid tissue of children appears to be more sensitive to ^131^I induced ablation than adults.

As in adults, we find that gland size influences therapy outcomes. In general, higher dosages per gm of thyroid tissue are needed with larger than smaller glands. Yet, with glands larger than 80 gm, ^131^I efficacy is low and is not recommended.

As in adults, when children are to be treated with ^131^I, ATDs should be stopped 3 to 5 days prior to treatment [90]. Patients are then placed on beta-blockers until T4 and/or free T4 levels normalize post-therapy. Whereas some clinicians restart ATDs after treatment with ^131^I, this is rarely required in children [4,62,90,91]. Thyroid hormone levels begin to decrease about 7 days after radioiodine therapy in children and continued ATD use can make it difficult to assess if post-treatment hypothyroidism is the result of ^131^I or the ATD.

Some centers give a fixed administered dosage of 10 or 15 mCi ^131^I to all children [91] rather than individually calculated administered activation. There are no studies comparing outcomes of fixed doses vs. calculated doses in children. In adults, the two different approaches lead to similar outcomes [92,93]; however, in children, a potential advantage of calculated vs. fixed dosing, is that it might be possible to use lower dosages of ^131^I if the administered dose is calculated.

Side effects of ^131^I therapy are unusual. Less than 10% of children will complain of mild tenderness over the thyroid in the first week after ^131^I therapy. This can be treated with either acetaminophen or non-steroidal, anti-inflammatory agents for 24 to 48 hrs [62,90].

There are rare reports of children with severe hyperthyroidism developing thyroid storm after ^131^I [67]. In general, these children were severely hyperthyroid when ^131^I was rendered. Thus, if T4 levels are >20 ug/dl (200 nmol/l) or freeT4 levels are >5 ng/dl (60 pmol/l), children should be treated with MMI until T4 and/or free T4 levels normalize before proceeding with ^131^I therapy [90]. It is important to recognize that most children with GD have been hyperthyroid for months prior to diagnosis; there is no need to rush to ^131^I therapy.

Following ^131^I, T3, T4 and/or free T4 levels should be obtained monthly. Because TSH levels may be suppressed for several months after the hyperthyroid state is corrected. Thus, TSH determination may not be useful post-therapy. Typically, hypothyroidism develops by 2 to 3 months after treatment [90,91]. When T4 levels fall below normal, levo-thyroxine is prescribed.

### Ophthalmopathy

The development of progression of ophthalmopathy following ^131^I in adults has been reported. However, unlike adults, children rarely develop severe ophthalmopathy and proptosis is mild.

Studies show that disease worsens in only a small percentage of children with GD, irrespective of therapy type. Of 87 children treated with ^131^I for GD at one center, proptosis improved in 90% of children, did not change in 7.5%, and worsened in 3% post-therapy [75,89]. In 45 children who had ophthalmopathy at the onset of treatment at another center, eye disease improved in 73% and worsened in 2% after one year or more of drug therapy [94]. After subtotal thyroidectomy in 80 children, eye disease was worsened in 9% [95], and was stable in 75% after total surgical thyroidectomy [95].

In adults, it has been shown that progression of ophthalmopathy can be prevented by treatment with prednisone for 3 months following ^131^I therapy [96]. Adjunctive prednisone therapy is not routinely recommended for the majority of children, as most do not have significant eye disease. The prolonged administration of prednisone is also associated with growth failure, weight gain and immune suppression. Nevertheless, prednisone may be useful for the child who has severe eye disease and will be treated with ^131^I.

### The risks of genetic damage with radioactive iodine

There is no evidence showing adverse effects to offspring of children treated with ^131^I. Birth defects were not higher in 500 offspring born to about 370 individuals treated with ^131^I for hyperthyroidism during childhood or adolescence [4]. Additionally, the rates of birth defects are not higher in children treated with 80–700 mCi of ^131^I for thyroid cancer, which are dosages that are much higher than those used for GD [97].

### Thyroid neoplasm risk with radioactive iodine

The thyroid gland is unique in its developmental sensitivity to malignancy after low-level radiation exposure [98-101]. There is an increased risk of thyroid cancer in individuals less than 20 years of age at the time of low-level thyroid irradiation, and the younger one is, the greater the thyroid cancer risk [98-100]. In contrast, individuals who are older than 20 years of age, do not exhibit an increased risk of thyroid cancer when exposed to low-level thyroid irradiation [98-101].

Importantly, the risk of thyroid neoplasms is greatest with exposure to low-level external radiation (0.1-25 Gy; ~0.09-30 uCi/gm) [98-102] and not with the higher dosages used to treat GD. At present, we are not aware of any cases of thyroid cancer that developed in pediatric patients treated with >150 uCi of ^131^I per gm of thyroid tissue for childhood GD that can be attributed to ^131^I therapy. Thus, it is important that low dosages be avoided.

### Non-thyroid cancer risks with radioactive iodine

Along with the risk of thyroid cancer, the potential influences of ^131^I therapy on other cancers must be considered since ^131^I therapy results in low-level, whole body radiation exposure. Several studies in adults have examined potential risks of ^131^I therapy for GD on cancers (Table 2). These studies have not revealed increased mortality or increased rates of cancer following ^131^I for GD [103-109].

**Table 2:** **Total cancer and cancer mortality related to **^**131**^**I therapy for hyperthyroidism in adults**

**Author**	**Date**	**Site**	**Sample**	**Outcome**	**Reference**
Ron	1998	US	23,020	No Effect***	[109]
Holm	1991	SW	10,000	No Effect**	[107]
Franklyn	1998	UK	7,209	No Effect	[104]
Flynn	2006	UK	3,888	No Effect	[103]
Metso	2007	FN	2,793	No Effect*	[108]
Franklyn	2005	UK	2,668	No Effect	[105]
Goldman	1982	US	1,762	No Effect	[106]

***Increase in thyroid CA with Nodular Disease.

**20% increase in stomach CA.

*15% Increase in stomach CA in Elderly Men with Nodular Disease.

In comparison with studies in adults, few studies have focused on outcomes of ^131^I therapy for childhood GD. The most extensive study of pediatric patients involved 36 year outcomes of 116 patients who were less than 20-years old when treated with ^131^I therapy for GD [110]. There was no evidence for increase cancer risk in this population.

The total-body radiation dose after ^131^I varies with age, and the same absolute dose of ^131^I will result in more radiation exposure in a young child than in an adolescent or adult [111,112]. Currently, we do not have dosimetry data on ^131^I use in pediatric patients with GD to assess total body exposure in pediatric patients. Based on phantom modeling, it is estimated that at 0, 1, 5, 10, 15 years, and adulthood, respective total body radiation doses will be 11.1, 4.6, 2.4 1.45, 0.90, and 0.85 rem (0.01 Sv) per mCi of ^131^I administered [111,112]. Based on the Biological Effects of Ionizing Radiation Committee V (BEIR VII) analysis of low-level, acute exposure to radiation [113], theoretical lifetime attributable risk of cancer mortality and all cancer incidence can be projected. Based on these theoretical calculations, we feel that it is prudent to avoid radioactive iodine therapy in children under 5 years of age and to avoid >10 mCi in patients less than 10 years old. Yet, these recommendations are based on theoretical concerns and not on hard data.

We recognize that there may be circumstances when ^131^I therapy is necessary for young children. The need for ^131^I in a young child may occur when the child develops a toxic reaction to an ATD, proper surgical expertise is not accessible, or the child is not a suitable surgical candidate.

## Surgery

The oldest form of definitive GD therapy is surgery, with the Nobel Prize in Physiology and Medicine awarded in 1909 to Koker for developments in this area. When surgery is considered, near total or total-thyroidectomy is indicated, as subtotal thyroidectomy is associated with a higher relapse rate [95]. Hypothyroidism is nearly universal in children and adults who undergo total thyroidectomy [95,114-116]. In comparison, after subtotal thyroidectomy, hyperthyroidism recurs in 10-15% of patients [95,114,115].

Surgery is preferred in children younger than 5 years when definitive therapy is needed AND can be performed by a skilled thyroid surgeon. In individuals who have large thyroid glands (>80 gm), the response to ^131^I is poor [63,117]. Thus, surgery is recommended for these patients.

In preparation for surgery, the patient should be rendered euthyroid. Typically, this is done by continuing MMI until T4 levels normalize. A week before surgery, iodine drops are started (5 to 10 drops, t.i.d.), which inhibits thyroid hormone production and causes the gland to become firm and less vascular, facilitating surgery.

Postoperatively, younger pediatric patients are at a higher risk for transient hypoparathyroidism than adolescents or adults [118]. To mitigate post-operative hypocalcemia, we treat children with 0.5 mcg of calcitriol, twice a day, for 3 days prior to surgery. Post-operatively, the calcitriol is weaned over 15 days (0.5 mcg bid × 5 days; 0.5 mcg qd × 5 days; 0.5 mcg qod x 5 days) [119]. Using this approach only 5% of patients require post-operative calcium infusions vs. 40% of patients without preoperative treatment [119].

### Complications of surgery

Acute complications that follow thyroidectomy include hemorrhage, hypocalcaemia, and recurrent laryngeal nerve paresis [118,120-123]. In children, rates from 0–6 years were 22%, from 7–12 years, 11%; and from 13 to 17 years, 11% [118]. These rates are higher than those in adults.

Complication rates are also related to the type of surgeon. When performed by pediatric surgeons, the complication rate for total thyroidectomy is approximately 15%. In comparison, the complication rate in children for high-volume thyroid surgeons (>30 thyroidectomies/year) is approximately 4%.

Considering these data, if local pediatric thyroid surgery expertise is unavailable, referral of a child with GD to a high-volume, thyroid surgery center with pediatric experience should be considered [124,125]. Very low complication rates for children undergoing the thyroidectomies for GD have been reported with this type of multidisciplinary model [119,124].

## Conclusions

Based on what we know about both the risks of different treatments and the pathogenesis of GD, we can be discriminating in our approach to therapy. To reduce the risks of treatment and to expedite cure, treatment should be guided by the patient’s age, the nature of the intrinsic autoimmune disease, and by expertise.

For children less than 5 years old, MMI should be considered as a first-line therapy. While radioactive iodine has been successfully used in this age group without an apparent increase in cancer rates [89,126], it may be wisest to defer radioactive iodine therapy until older.

Because young children are less likely to have remission on drug treatment vs. older children [42,46], prolonged drug therapy may be necessary. Assuming there are no toxic effects, continuing MMI is sensible until the child is old enough for ^131^I. If reactions to medication develop, or there is the desire to avoid prolonged drug use, thyroidectomy or ^131^I can be considered. Fortunately, less than 5% of children with GD present at 5 years or younger [127].

It is important to emphasize that when ATDs are used, only MMI should be prescribed. PTU use should be restricted to special circumstances when neither prompt surgery nor ^131^I treatments are possibilities in a patient who has developed a toxic reaction to MMI, and ATD therapy is required. In this setting, the use of PTU should be short-term.

Fifteen percent of children with GD will present between 6 years and 10 yrs of age [127]. It is reasonable to consider MMI therapy as a first line measure for this age group. As 10 years of age is approached, either drug therapy or radioactive iodine can be considered as an initial therapy.

Children who are 10 years and older account for 80% of the pediatric GD cases. Radioactive iodine or MMI can be considered as first line treatment options for this age group. TRAb levels and thyroid size may be predictive of remission rates. The presence of low TRAb levels and a small thyroid is suggestive of the possibility of spontaneous remission after at least one year of medical therapy. Yet, if the thyroid is large and TRAb levels are high, the odds of spontaneous remission are low [47,128,129].

For those patients who have normal TRAb levels and a small thyroid, it is reasonable to treat for one to two years and stop the drug when clinical remission is achieved. If relapse occurs, medical treatment can be resumed or an alternative form of therapy chosen. For patients with elevated TRAb levels and a large thyroid size, it is less likely that remission will occur after medical therapy. Thus, definitive treatment soon after euthyroidism is achieved can be considered.

When radioactive iodine is used, it is important that the appropriate dosage be administered. The objective of radioactive iodine therapy in pediatric patients should be to ablate the thyroid gland and achieve hypothyroidism. The risk of thyroid cancer will be very small, if present at all, if no thyroid tissue remains, To achieve this objective, doses of ^131^I >150 uCi/gm of thyroid tissue are needed, with higher doses needed for larger glands.

Finally, regardless of the treatment option selected, careful follow up is essential for all patients treated for GD. Long-term follow-up should include, once or twice a year, regular examination of the thyroid gland and measurement of circulating levels of thyroid hormones.

Selecting a treatment approach for childhood GD can be challenging and personal decision. It is essential that physicians discuss the advantages and risks of each therapeutic option to help the patient and family select the treatment plan they feel comfortable with.

## Competing interests

The author declares that there are no competing interests.
